# Impact of Model Selection and Conformational Effects on the Descriptors for In Silico Screening Campaigns: A Case Study of Rh-Catalyzed Acrylate Hydrogenation

**DOI:** 10.1021/acs.jpcc.4c01631

**Published:** 2024-05-02

**Authors:** Margareth S. Baidun, Adarsh V. Kalikadien, Laurent Lefort, Evgeny A. Pidko

**Affiliations:** † Inorganic Systems Engineering, Department of Chemical Engineering, Faculty of Applied Sciences, Delft University of Technology, Van der Maasweg 9, 2629 HZ Delft, The Netherlands; ‡ Discovery, Product Development and Supply, Janssen Pharmaceutica N.V., Turnhoutseweg 30, 2340 Beerse, Belgium

## Abstract

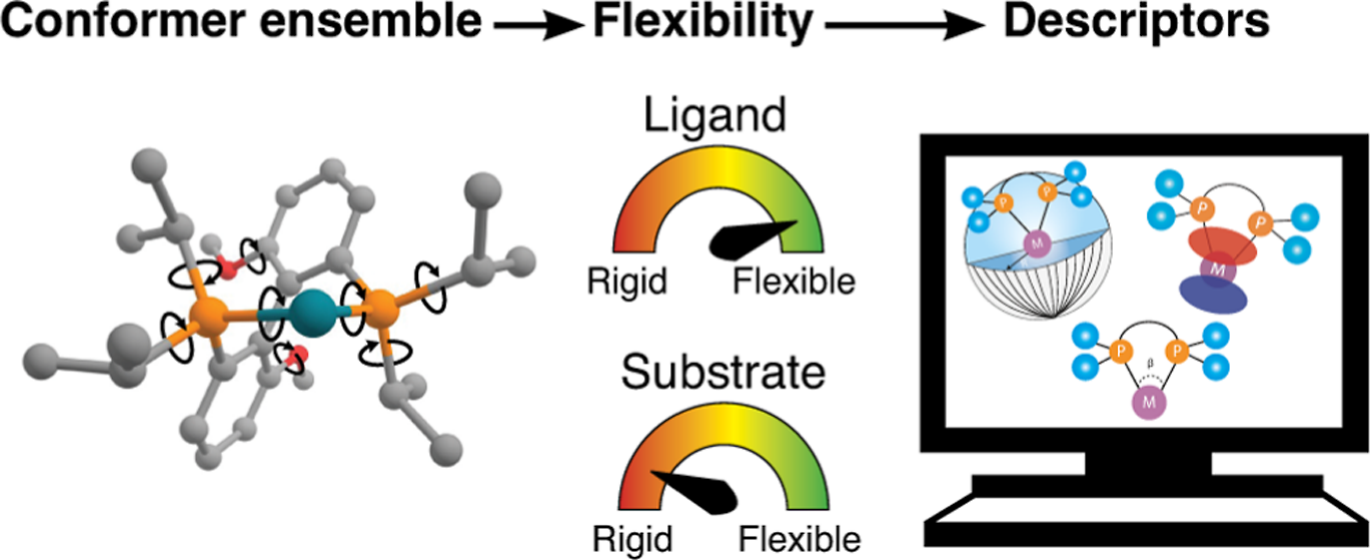

Data-driven catalyst design is a promising approach for addressing the challenges in identifying suitable catalysts for synthetic transformations. Models with descriptor calculations relying solely on the precatalyst structure are potentially generalizable but may overlook catalyst–substrate interactions. This study explores substrate-specific interactions in the context of Rh-catalyzed asymmetric hydrogenation to elucidate the impact of substrate inclusion on the catalyst structure and on the descriptors derived from it. We compare a catalyst–substrate complex with methyl 2-acetamidoacrylate as a model substrate with the generic precatalyst structure involving a placeholder substrate, norbornadiene, across 11 Rh-based catalysts with bidentate bisphosphine ligands. For these systems, a full conformer ensemble analysis reveals an intriguing finding: the rigid substrate induces conformational freedom in the ligand. This flexibility gives rise to a more diverse conformer landscape, showing a previously overlooked aspect of catalyst–substrate dynamics. Electronic descriptor variations particularly highlight differences between substrate-specific and precatalyst structures. This study suggests that generic precatalyst-like models may lack crucial insights into the conformational freedom of the catalyst. We speculate that such conformational freedom may be a more general phenomenon that can influence the development of generalizable predictive models of computational TM-based catalysis.

## 1 Introduction

In the pursuit of more sustainable and efficient chemistry, finding suitable catalysts to drive homogeneous chemical reactions is one of the main challenges. Particularly in the pharmaceutical industry, the precision of chemical synthesis is essential to producing stereospecific compounds.^1,2^ Asymmetric hydrogenation is a powerful tool to ensure stereoselectivity, with various transition-metal complexes achieving enantiomeric excess (ee) values above 99%.^3−10^ The key to the catalyst’s success lies in selecting appropriate ligands to tune the reactivity and guide the chemical conversion along the desired pathway.^11−13^ However, since the relationship between ligand structure and catalyst performance is not straightforward, identifying optimal ligands within the broad range of chemical possibilities remains challenging.

With the vast rise of computational resources,^14−18^ computational chemistry emerges as a promising tool for chiral ligand design.^19^ Different approaches to data-driven models for catalysis exist, categorized as being based on the reaction-specific mechanism,^20−23^ or mechanism-agnostic model structures.^12,24−26^ One mechanism-agnostic approach involves computing 3D descriptors to characterize catalyst structures.^12,27−29^ These descriptors aim to identify ligands that optimize certain attributes, such as reactivity or selectivity, often measured in terms of conversion or ee. Singh et al.^30^ base their descriptor calculations on the ligands of different binaphthyl catalyst families to study the hydrogenation of different substrates bearing C=C and C=N bonds. Zahrt et al.^31^ focus on predicting selective catalysis outside the range of selectivity values observed in the training data, with descriptor calculations based on a set of chiral phosphoric acids as model catalysts. Dotson et al.^12^ calculate the descriptors based on a [ligand]PdCl_2_ model system to predict both reactivity and selectivity. In these approaches, the substrate is included in the form of separate molecular parameters, thus considering the substrate separately from the precatalyst structure. The main hypothesis is that descriptors based on such a precatalyst structure, not including the substrate, capture essential catalyst characteristics and can adequately represent the performance.

Relying solely on the precatalyst structure may not be sufficient to predict and understand the catalytic behavior. Already in the 1980s, it was suggested that valuable insights into enantioselectivity could be derived from the reversible substrate coordination, which is suggested to dictate stereoselection.^32−36^ Moreover, the lowest energy conformer of the precatalyst structure is often taken as particularly important, which may not be a valid assumption. Recent studies challenge the focus on the lowest energy conformer, aligning with the ‘lock-and-key hypothesis’.^37^ These studies suggest that catalyst flexibility, reflected in the existence of a conformer ensemble, enables adaptable chiral pockets, enhancing selectivity.^38−40^ Acknowledging the significance of the substrate and catalyst flexibility for selectivity, it becomes plausible that structural variations induced by the substrate itself play a crucial role. This catalyst flexibility may introduce variations in descriptor values that are important to consider when predicting reactivity and selectivity in descriptor-based catalyst design approaches.

**Figure 1:**
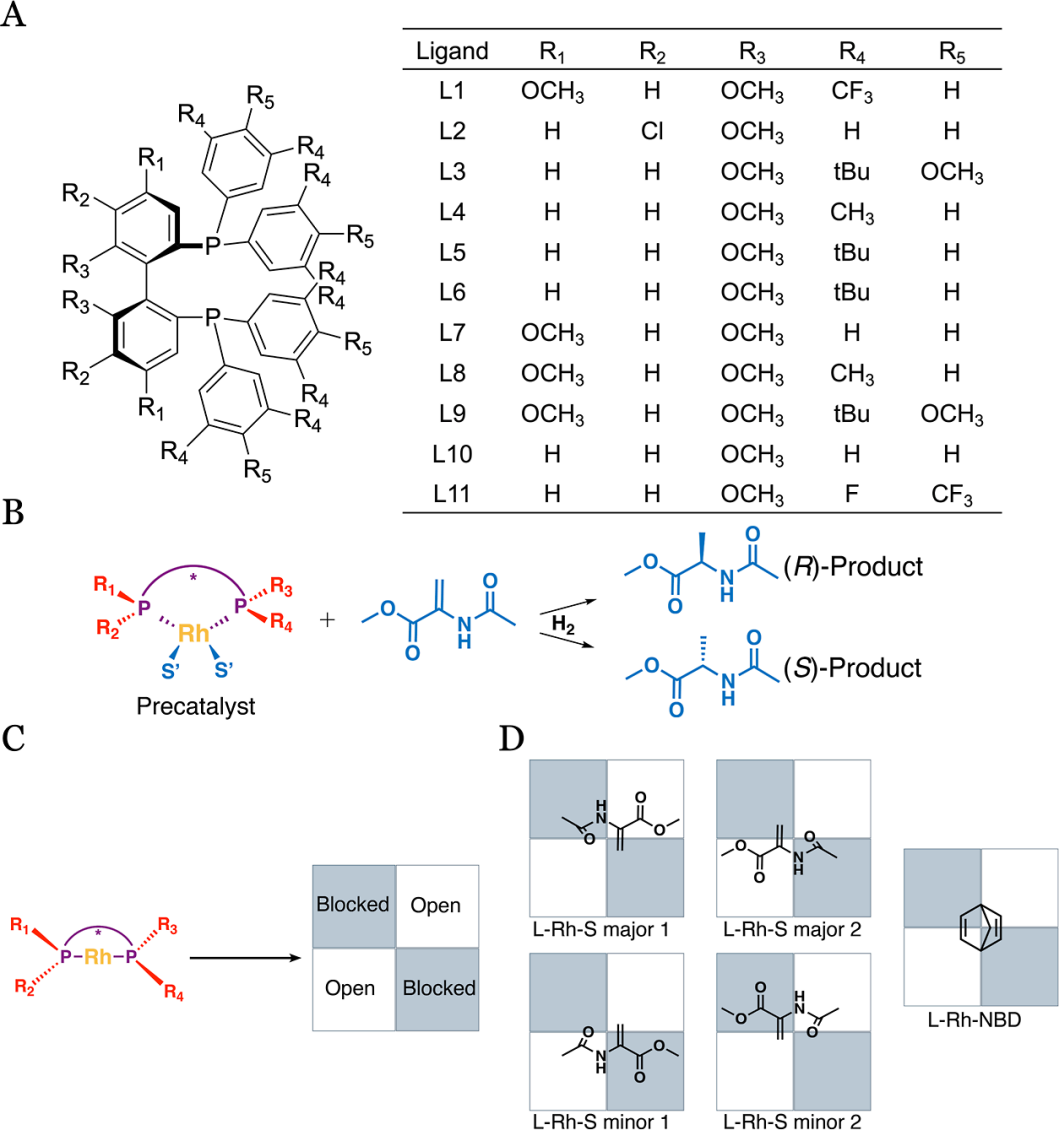
Backbone of the 11 studied ligands with their respective substituents (A) for Rh-catalyzed hydrogenation of methyl 2-acetamidoacrylate (denoted as S) to the (*S*)- and (*R*)-products (B). Ligand groups lead to two open and two blocked quadrants (C), yielding four different coordination modes for S and one for NBD (D). According to the quadrant diagram, two S coordinations are less hindered, and two are more hindered coordinations, termed the major and minor coordinations, respectively.

Hence, the primary objective of the present work is to answer the following research question: can we quantify the effects of the specific substrate on catalyst structure and descriptor values compared to those of the precatalyst with a model substrate? To address this question, the asymmetric hydrogenation reaction of methyl 2-acetamidoacrylate (referred to as S) with 11 Rh-based model catalysts is computationally investigated. These catalysts are referred to as L-Rh-S, with L being 11 different bidentate bisphosphine ligands (Figure 1A). Four coordination possibilities of S to the metal center are explored following Knowles quadrants^32^ (Figure 1C). The L-Rh-S complexes are compared to the generic precatalyst structures with a model diene ligand, norbornadiene (NBD), referred to as L-Rh-NBD. The model ligand serves as a placeholder, fixing the ligand geometry in such a way that it can be correlated to the preferred binding of the substrate. A comprehensive conformational search is conducted on all L-Rh-S and L-Rh-NBD structures to establish the effect of the substrate on catalyst flexibility, followed by Boltzmann-averaged descriptor calculations to assess the effect of the conformer ensembles on substrate-specific descriptors.

The subsequent sections of the paper are organized as follows: the methods section outlines the applied workflow for structure generation, conformer search, structure comparison, and subsequent analyses. The results section starts with the conformer search outcomes of L-Rh-S compared to those of L-Rh-NBD. This investigation aims to study the influence of the specific substrate on the existence and characteristics of a conformer ensemble. Next, a detailed analysis of the catalyst flexibility is presented, including a separate evaluation of the ligand and substrate contributions to the structural flexibility in a conformer ensemble. Finally, the influence of a conformer ensemble on structural and electronic descriptors is shown. A direct comparison between L-Rh-S and L-Rh-NBD structures provides insights into how the specific substrate alters the descriptor values. To close, we derive some conclusions from these results regarding the effect of the specific substrate on structure and descriptor values, challenging conventional precatalyst-based approaches.

## 2 Computational Methods

**Figure 2:**
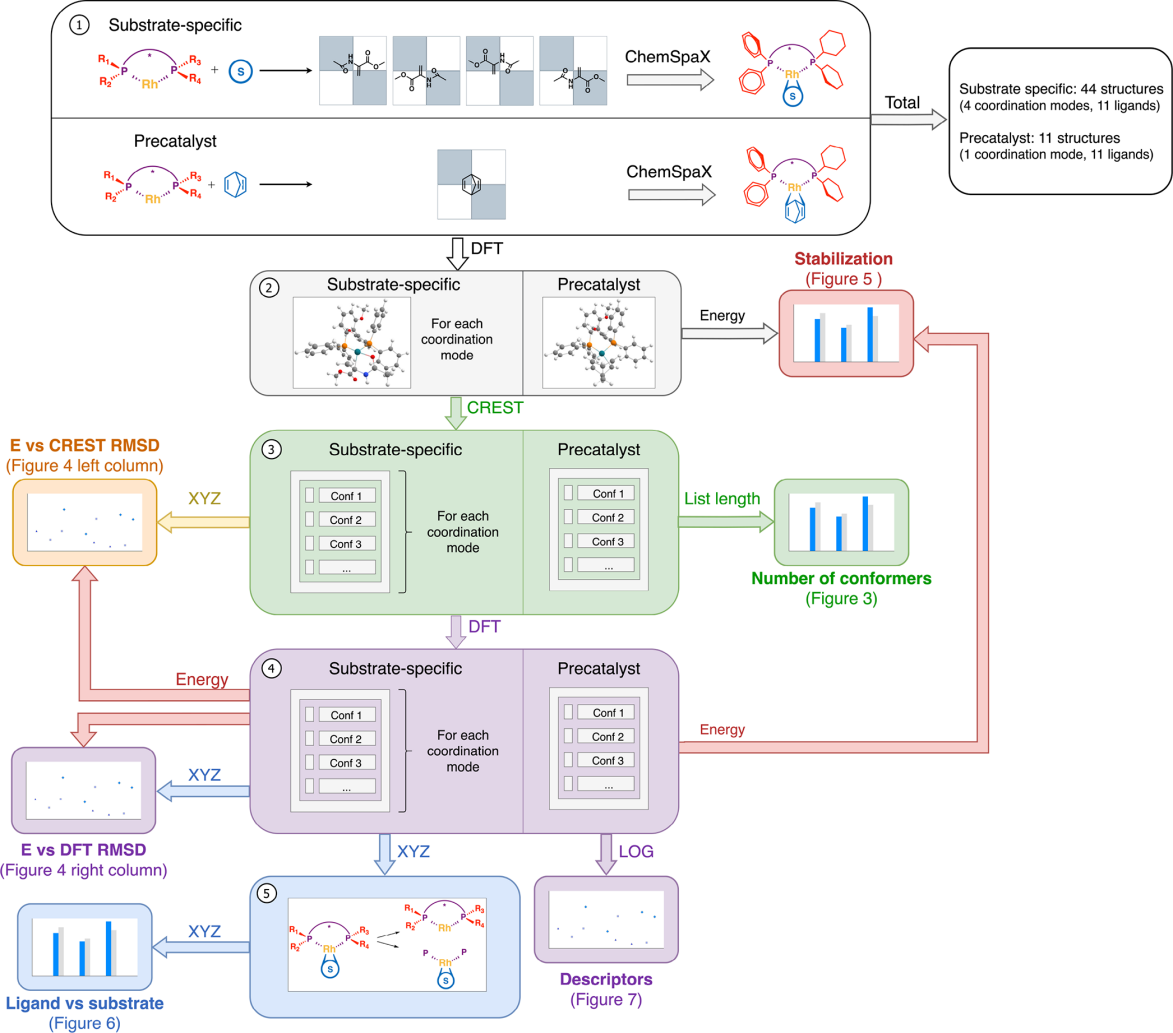
Summary of the applied workflow. Entire workflow, shown as an example for one ligand, is employed on all 11 ligands. In step 1, unique ligands in Rh complexes featuring different substrate coordination modes are generated. In step 2, these structures are DFT-optimized, followed by a conformer search in step 3. All generated conformers are DFT-optimized in step 4. In step 5, the DFT-optimized structures are separated into a substrate and ligand part for individual analysis. Workflow shows how the methodological steps are connected to the figures in the remainder of the paper.

For this study, we generated substrate-specific and precatalyst structures, performed conformer search and geometry optimization, and compared the conformers based on their energy, structure, and descriptor values. The workflow for these steps is illustrated in Figure 2. An example is shown for one ligand, but the same workflow was applied to all other ligands. The figure connects the methodological steps with the corresponding figures in the results section. Below, the workflow steps are discussed, followed by specific computational chemistry details.

### 2.1 Workflow

#### 2.1.1 Ligand Generation

A total of 11 Rh-based catalysts with bidentate bisphosphine ligands were studied in this work, with NBD and S coordinated to the metal center. All of the studied ligands have a common backbone structure (Figure 1A). With this backbone structure, a total of five catalyst–substrate structures were built manually: two with S in the major coordination, two with S in the minor coordination, and one involving NBD (Figure 1D), termed L-Rh-S major, L-Rh-S minor, and L-Rh-NBD, respectively. Specific substituents, shown in Figure 1A, were introduced into these five structures to generate the unique ligands. This functionalization process was done using ChemSpaX,^41^ an open-source software developed by our group, designed to automate the placement of substituents on ligands within a 3D space. This process is shown in step 1 of Figure 2, resulting in a total of 44 unique structures with S coordinated to the metal center and 11 structures involving NBD instead.

#### 2.1.2 Geometry Optimization and Conformer Search

The generated structures underwent DFT geometry optimization (see Section 2.2 for details), yielding four structures per ligand with different S coordinations alongside one structure featuring NBD coordination (step 2). Conformational exploration was conducted on the 55 DFT-optimized structures, as shown in step 3, with subsequent DFT-geometry optimization on all generated conformers (step 4). Specific details regarding the conformer searches are described in Section 2.2. Within each conformer set, the DFT-based energies of the conformers (data in step 4) were compared to the DFT energies of the CREST^42,43^ input structures (data in step 2) to assess the degree of stabilization achieved through conformer search. Furthermore, the geometry-optimized conformers were compared to each other to assess the variability within the generated conformer ensembles. This was done by analyzing the DFT-optimized energies, together with structural differences. The structural differences were calculated on the structures both before (data in step 3) and after DFT optimization (data in step 4), with details of the structural difference calculations presented below.

#### 2.1.3 Structural Differences

Structural variations were assessed by calculating the minimal root-mean-square deviation (rmsd)^44^ between conformers. The structural variations were computed relative to the conformer with the lowest DFT-based energy within the respective conformer set. All hydrogen atoms were ignored in the calculation of rmsd values with the option -no-hydrogen. These structural difference calculations were performed in three stages: on the conformer structures generated by CREST (data in step 3), on the DFT-optimized conformer structures (data in step 4), and on isolated ligand and substrate components (vide infra, data in step 5).

#### 2.1.4 Ligand vs Substrate

Following DFT optimization, step 5 illustrates how each conformer structure is separated into a substrate part and a ligand part. The ligand part comprises the atoms of the bidentate ligand and the metal center, while the substrate part consists of the substrate molecule (either S or NBD), the metal center, and the two P donor atoms. The inclusion of donor atoms aimed to establish an orientation reference for the substrate. Subsequently, structural difference calculations were performed on the isolated ligand and substrate parts.

#### 2.1.5 Descriptor Calculation

Descriptor calculations were performed on all DFT-optimized conformers (data in step 4) using the in-house developed method OBeLiX (Open Bidentate Ligand eXplorer).^45^ OBeLiX is an automated and reproducible workflow for computing transition metal-based catalyst descriptors. The workflow accommodates descriptor calculations from xyz files, Gaussian^46^ log files, or CREST output folders, with this work specifically relying on Gaussian log files. Using Morfeus^47^ and cclib,^48^ the workflow computes 75 descriptors categorized as steric, geometric, electronic, or thermodynamic.

This work focuses on a subset of five descriptors: NBO charge on Rh, NBO charge on the donor atoms of the ligand, buried volume on Rh, buried volume on the donor atoms of the ligand, and the HOMO–LUMO gap. These calculated descriptors were Boltzmann-weighted and averaged. With the goal of quantifying populations within the given substrate coordination ensembles, we calculated the Boltzmann factors separately for L-Rh-S major, L-Rh-S minor, and L-Rh-NBD structures. The Boltzmann weights were determined using the formula . In this formula, *w* represents the weight, *E* is the energy obtained from DFT calculations, *k*_b_ is the Boltzmann constant, *T* is the temperature (289 K), and *E*_min_ is the corresponding DFT-based energy of the lowest energy conformer of L-Rh-S major, L-Rh-S minor, or L-Rh-NBD. The resulting weights were used to calculate the Boltzmann-averaged descriptor values, along with standard deviation values.

### 2.2 Computational Method Details

All geometry optimization calculations were performed using the Gaussian 16 C.01 suite.^46^ The calculations were executed at the PBE0^49^-D3(BJ)^50^/def2-SVPP^51^ level of theory. The chosen combination of functional and basis set has shown reliable results for similar transition metal-based complexes, accompanied by low computational costs.^41,52^ The nature of each stationary point was confirmed via a frequency analysis. In cases where imaginary frequencies were present, these were removed with the pyQRC Python package,^53,54^ followed by an additional geometry optimization. All calculations were carried out in the gas phase. A Natural Population Analysis (NPA) was performed using the NBO program version 3.1^55^ as implemented in Gaussian.

Conformer search was done using the CREST software (version 2.12)^42,43^ with xtb (version 6.6.1)^56^ optimization. To efficiently screen the configurational space and find low-lying conformers, CREST makes use of MD simulations with a bias potential.^43^ Generated conformer ensembles were selected within 6 kcal/mol of the lowest energy conformer, and calculations were carried out at the GFN2-*x*TB//GFN-FF level of theory. We evaluated the conformer search for a set of representative ligand structures with various GFN*n*-*x*TB methods, with the results in the Supporting Information (Section S1.1) showing that the GFN2-*x*TB//GFN-FF method was the most suitable option in terms of computational cost and avoiding structural artifacts. Throughout this work, conformers generated with this method are referred to as “CREST” conformers. To preserve the chirality of the ligand, the aromatic rings on the chiral axis were fixed during xtb optimization and conformer search. Additional details about chirality preservation are provided in the Supporting Information (Section S1.2). The −noreftopo option was employed to disable topology checks before conformer search, ensuring proper treatment of transition-metal complexes. Following the conformer search, all generated conformers were checked on chirality, and L-Rh-S structures were checked on maintaining the initial substrate coordination mode. Two conformers from the L11-Rh-S major 1 conformer set were removed due to the rotation of the substrate to another coordination mode.

## 3 Results and Discussion

The results section is divided into two parts. The first part addresses the conformer search outcomes of L-Rh-S compared with L-Rh-NBD. The second part delves into the individual evaluation of ligand and substrate contributions to the configurational freedom, followed by a discussion of a set of descriptors calculated on the conformer ensembles. In the following, structural differences within a given metal-ligand complex are termed the “flexibility” of the system.

### 3.1 Conformer Search

**Figure 3:**
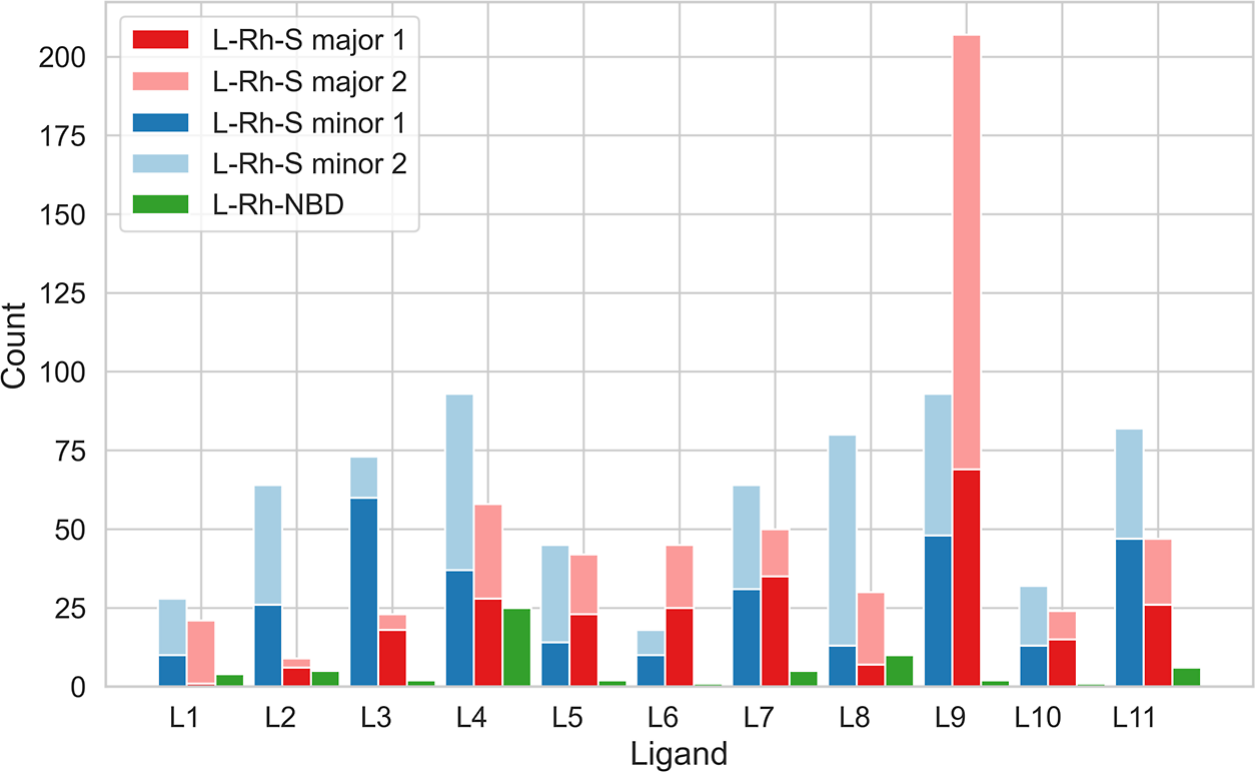
Number of conformers from CREST output for L-Rh-S major, L-Rh-S minor, and L-Rh-NBD. For L-Rh-S major and minor, two major and two minor coordinations are considered, resulting in two conformer sets each, indicated by “1” and “2”.

A comparative study of conformer ensembles involving S and NBD was performed on a total of 44 L-Rh-S and 11 L-Rh-NBD complexes. S can coordinate in four ways to the metal center, with two major and two minor coordinations (Figure 1D), yielding four input structures for the conformer search and four separate conformer ensembles. NBD, having a single coordination mode, results in one input structure and conformer ensemble instead of four. Figure 3 shows the total number of conformers generated for the L-Rh-S and L-Rh-NBD complexes. Two major and two minor conformer ensembles are stacked on top of each other, and the cumulative major and minor counts are shown next to each other.

Across the ligands, we can first consider the two L-Rh-S major and two L-Rh-S minor conformer ensembles separately. For instance, L-Rh-S major for L3 exhibits a significant difference in conformer count between S major 1 and S major 2, with 18 and 5 conformers, respectively. This discrepancy is evident in ligands L1, L8, and L9 as well despite having the same substrate coordination mode. Similar observations can be made by comparing two minor conformer ensembles within each ligand, best visualized in the conformer ensembles of L3 and L8.

Second, we can compare L-Rh-S conformer ensembles to L-Rh-NBD ensembles for each ligand. A consistent observation is that at least one major and one minor set of conformers involving S contains significantly more conformers compared to the ensemble of the complex with NBD. This implies that the L-Rh-NBD precatalyst structure is rather rigid while substrate coordination induces flexibility, resulting in a larger conformer ensemble. However, merely counting the generated conformers itself does not provide insight into ensemble diversity or the origin of catalyst flexibility.

**Figure 4:**
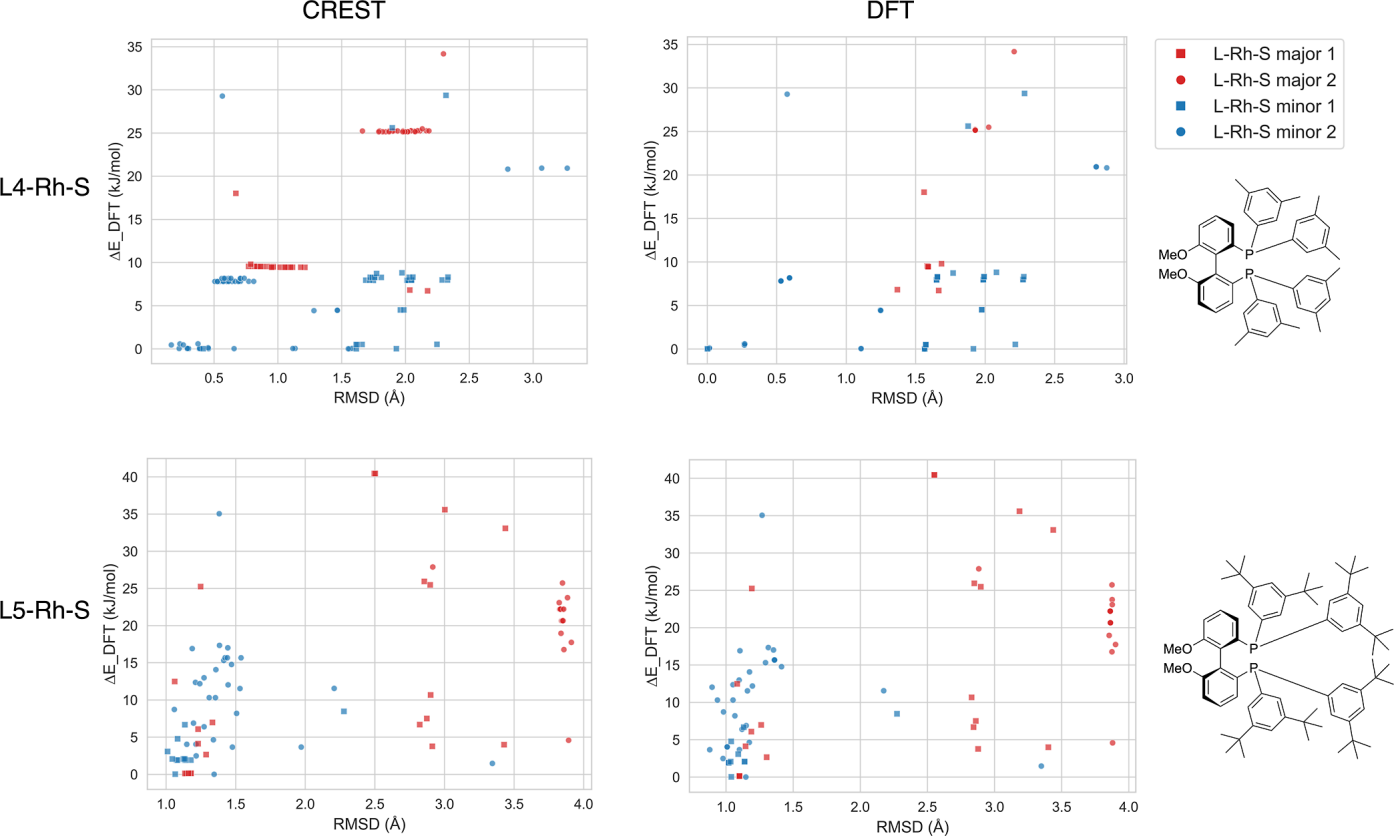
Relative energies plotted against structural variations (rmsd) for the conformer ensembles of L4-Rh-S (upper row) and L5-Rh-S (lower row). rmsd values are obtained from CREST (left column) or DFT structures (right column) and the relative energy is DFT-based in all four subplots. Within each conformer ensemble, the conformer with the lowest DFT-based energy is taken as a reference point for the Δ*E* and rmsd values.

To assess the diversity of the generated conformers, the structural differences are compared before and after DFT optimization, as illustrated in Figure 4. The *y*-axis in each subplot reflects the DFT-based energy differences, while the *x*-axis represents the structural differences, both relative to the lowest energy conformer within each conformer set. The structural differences are represented by the rmsd value calculated on either CREST-based (left column) or DFT-based (right column) conformer structures. The upper and lower rows show the subplots regarding L4-Rh-S and L5-Rh-S, respectively. These ligands are chosen to be analyzed in detail as illustrative cases, with similar analyses for ligands L1–L3 and L6–L11 presented in the Supporting Information (Section S2).

When comparing the rmsd values of the DFT-optimized structures to the values of the CREST-based structures, one can see whether the conformers identified by CREST remain true, with distinct minima after DFT optimization. For L5-Rh-S (subplots in the lower row), minimal differences between the left and right subplots indicate that the structural differences identified by CREST are preserved after DFT optimization. For this ligand, a total of 87 conformers identified by CREST converged to 62 distinct conformers after DFT optimization. L4-Rh-S (subplots in the upper row) shows a contrasting picture. While CREST reveals distinct conformers close in rmsd values, DFT optimization converges many of these conformers to the same minima. Here, 151 conformers identified by CREST converged to 35 distinct conformers. These findings underscore the value of DFT optimization for a comprehensive understanding of the system’s flexibility.

Analyzing the structural differences after DFT-optimization (right column) reveals many structures with varying rmsd values but minimal energetic differences. For instance, the DFT-optimized structures of L4-Rh-S (upper right subplot) contain several structures around Δ*E* of 0 kJ/mol but with vastly different rmsd. These instances pose a challenge in selecting conformers for descriptor-based catalyst design. Energetically identical structures with significant structural variations may yield diverging descriptor values, influencing the predictions. Additionally, conformer ensembles with varying energy values but similar rmsd values can be found, as well. An example is the conformers in L5-Rh-S with rmsd values around 4 Å and Δ*E* values spreading from 5 to 26 kJ/mol. These findings emphasize the importance of careful conformer consideration, especially given that a DFT uncertainty as low as 5 kJ/mol can invert predicted enantioselectivity trends.^57^ A similar analysis for ligands L1-L11 with NBD coordination is presented in the Supporting Information, highlighting that the majority of generated conformers exhibit Δ*E* values below 5 kJ/mol with a few outliers. An intriguing insight emerges from these observations: the inability of the precatalyst structure to capture the system’s flexibility, translating into fewer conformers with less variability.

**Figure 5:**
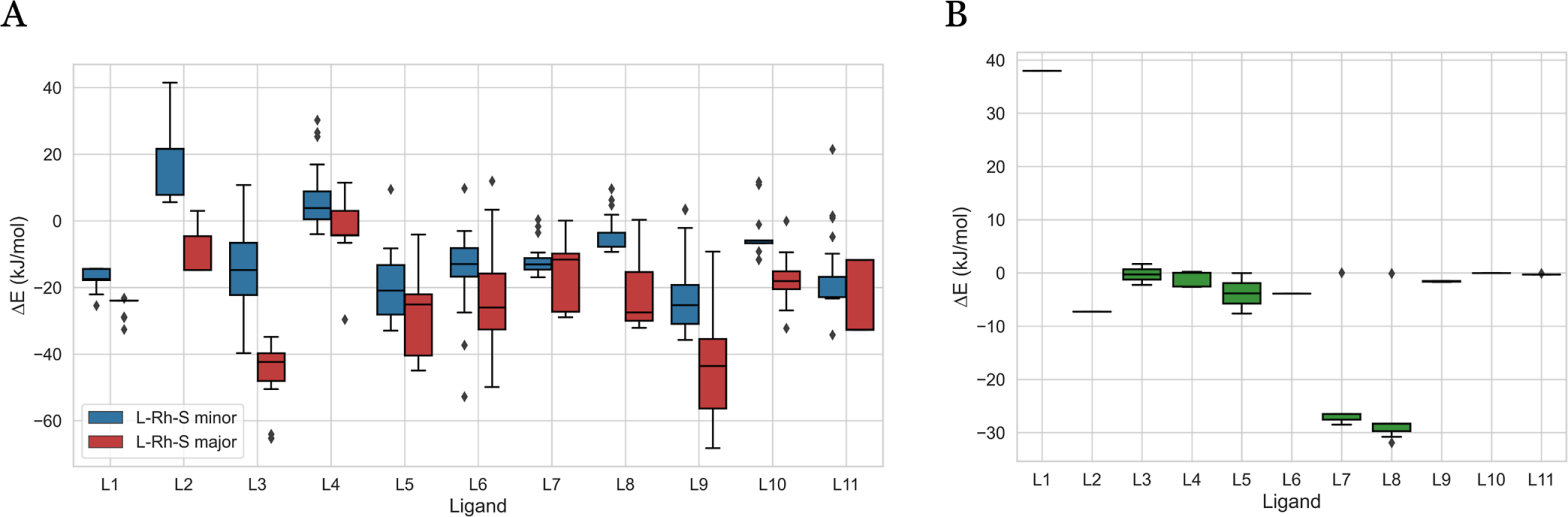
Energy range of the conformer ensembles after DFT optimization per ligand for L-Rh-S major and L-Rh-S-minor (A) and L-Rh-NBD (B). Per conformer ensemble, the DFT-based energy of the structure used as CREST input is chosen as the baseline.

Conformer search is not only useful for generating a conformer ensemble, but it is also essential in locating the global minimum.^42^Figure 5 shows the energy distribution within each ligand’s conformer ensemble relative to the energy of the CREST input structure. Except for L2-Rh-S minor in Figure 5A, conformer search with CREST successfully identifies conformers significantly lower in energy than the original structure on which conformer search was performed, surpassing 60 kJ/mol in some cases. With the exception of L1, this trend persists in Figure 5B for L-Rh-NBD, with CREST identifying conformers that are significantly lower in energy. In addition, the L-Rh-NBD conformers generally exhibit lower energies compared to the original structure, whereas the L-Rh-S conformer sets contain structures with both higher and lower stability in comparison to the original structure.

Comparing the number of generated conformers with the degree of energy minimization in the conformer ensembles offers insights into whether a high number of generated conformers corresponds to more significant stabilization. First, we can compare L-Rh-S major and L-Rh-S minor within each ligand. Taking L3 as an example from Figure 5A, L-Rh-S major exhibits a greater stabilization than L-Rh-S minor, despite the latter having a higher total number of conformers (Figure 3). This trend holds for 9 out of 11 ligands, where the coordination with a lower number of conformers exhibits greater stabilization than the coordination with a higher number of conformers, with the exception of L9 and L11. Second, we can compare all conformer ensembles across the ligands. For instance, L4-Rh-S minor is stabilized by approximately 5 kJ/mol despite the discovery of nearly 100 conformers. Conversely, L9-Rh-S major is the most stabilized structure, correlating with the highest number of conformers. This indicates that the trend of high stabilization with a low number of conformers does not hold true across all ligands.

A similar comparison can be drawn between the number of generated conformers and the energy range within the generated conformer ensembles. This analysis aims to discern whether the number of conformers offers insights into energetic variations in a conformer set. However, no consistent trend emerges across the ligands. While L9-Rh-S major displays a large number of conformers spanning from −68 to −9 kJ/mol, L4-Rh-S minor demonstrates the opposite. The large number of conformers results in a narrow energy range with a few outliers. This is corroborated by the top two subplots of Figure 4, where numerous conformers identified by CREST converge after DFT optimization to structures within a narrow range of 10 kJ/mol with a few outliers. These findings underscore the intricate relationship among energy minimization, energetic variability, and the number of conformers obtained with CREST.

### 3.2 Catalyst Flexibility

The previous section delved into the generation of extensive conformer ensembles with the inclusion of the specific substrate, revealing significant differences in both energy and structure. This section explores catalyst flexibility in detail. First, we disentangle the separate contributions of the ligand and substrate to the structural variations within the conformer ensembles. Next, the impact of these structural differences on a set of descriptors is examined.

#### 3.2.1 Ligand vs Substrate Contributions

**Figure 6:**
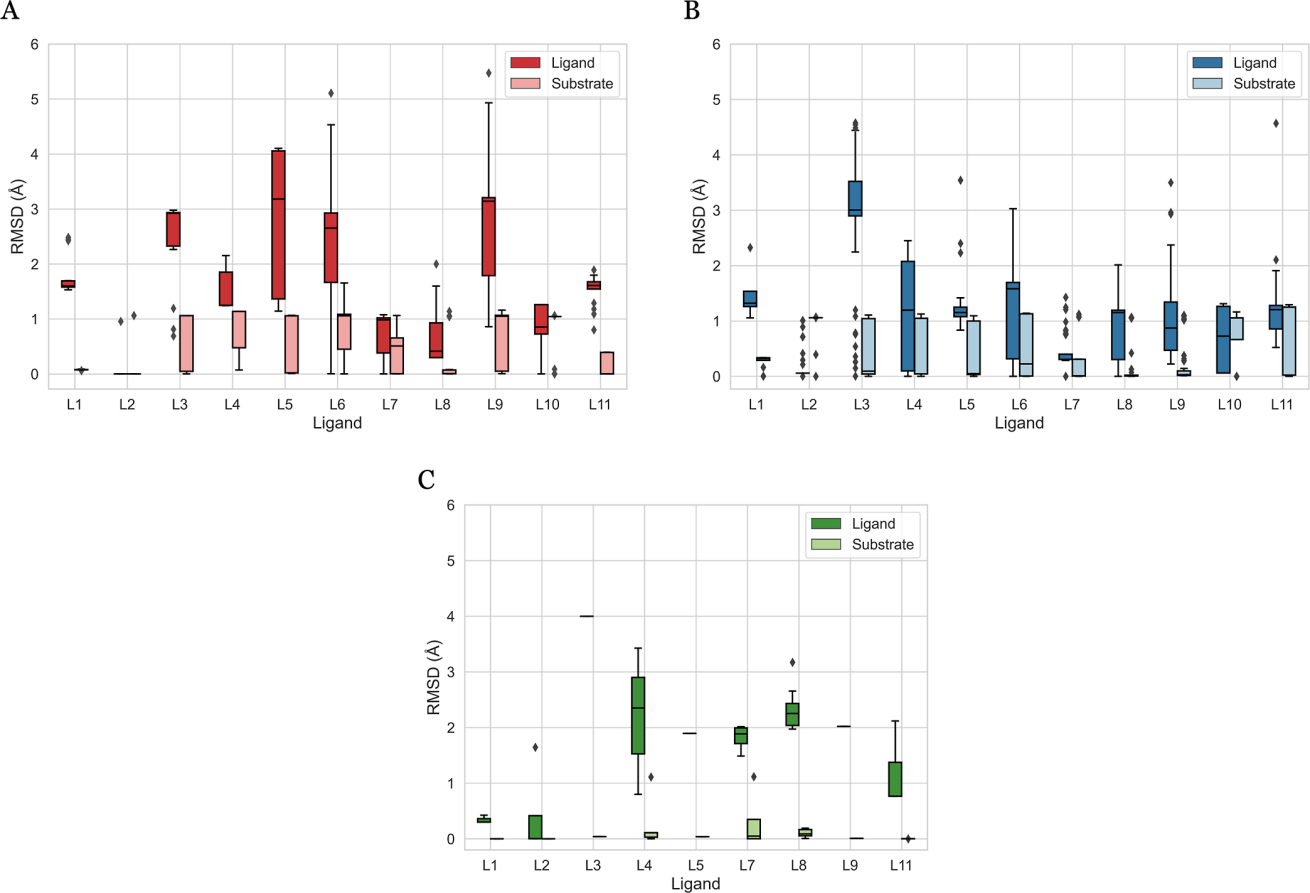
rmsd range of the conformer ensembles after DFT optimization per ligand for L-Rh-S major (A), L-Rh-S minor (B), and L-Rh_NBD (C) complexes. Within each conformer set, the ligand and substrate are isolated and the rmsd values of the ligand and substrate part are calculated relative to the conformer with the lowest DFT-based energy.

To discern the separate contributions of the ligand and substrate to structural variations within one metal–ligand system, the DFT-optimized structure of each conformer is separated into a ligand part and a substrate part, as outlined in the methods section. For each conformer, the structural differences of the substrate and ligand are calculated relative to the respective parts of the lowest energy conformer within the corresponding conformer set. These structural differences are represented by the rmsd values on the *y*-axis in Figure 6A (L-Rh-S major), B (L-Rh-S minor), and C (L-Rh-NBD) for each ligand.

The substantial increase in conformers introduced by the specific substrate compared with NBD (Figure 3) implies that the substrate induces conformational variation. The origin of this flexibility, from either the ligand or the substrate, is clarified by examining the individual rmsd contributions in Figure 6. The substrate rmsd value rarely exceeds 1 Å across different ligands and major/minor coordinations, indicating a high degree of rigidity. A similar trend is observed when the NBD is coordinated (Figure 6C), where substrate displays high rigidity with consistently low rmsd values.

Contrasting to the consistently low substrate rmsd, the ligand exhibits broad rmsd ranges, reaching values up to 5 Å. These findings suggest that the ligand dynamically adapts to the rigid substrate. This ligand flexibility is also seen in the L-Rh-NBD structures, although to a lesser extent, given the generation of fewer conformers when NBD is involved. The comparison to NBD suggests that the specific substrate induces flexibility in the ligand. As an asymmetric structure, S may induce preferential orientations of the ligand to optimize noncovalent interactions. In contrast, the highly symmetric NBD structure does not lead to specific orientation preferences for the ligand. The substrate-induced flexibility yields a higher number of conformers in L-Rh-S complexes compared to that in L-Rh-NBD, even though the substrate exhibits rigidity in both cases. These findings on ligand flexibility support the observations of Crawford and Sigman, where the ligand’s adaptability is suggested to stabilize intermediates and transition states throughout the catalytic cycle.^38^

The alignment between ligand flexibility and conformer diversity gains support when comparing the data in Figure 6 to those in Figure 4. Taking L5 as an example, Figure 6A,B reveals consistent substrate rigidity in both the L-Rh-S major and L-Rh-S minor structures. However, there is a distinctive difference in ligand flexibility. L-Rh-S major exhibits a broad range of rmsd values (1.14 to 4.10 Å), while L-Rh-S minor demonstrates a narrower rmsd range (around 1.2 Å) with some outliers. This observation aligns with the structural differences after DFT optimization for L5-Rh-S in Figure 4 (lower right subplot). Here, L-Rh-S minor points are clustered within a narrow rmsd range with a few outliers, while L-Rh-S major points are spread across a broader range of rmsd values. Together, these findings strengthen the hypothesis that structural variability within a conformer set is primarily driven by ligand flexibility. The detailed analysis of the origin of energy differences within the conformer ensembles is presented in Section S3 of the Supporting Information.

#### 3.2.2 Effect of Flexibility on Descriptors

**Figure 7:**
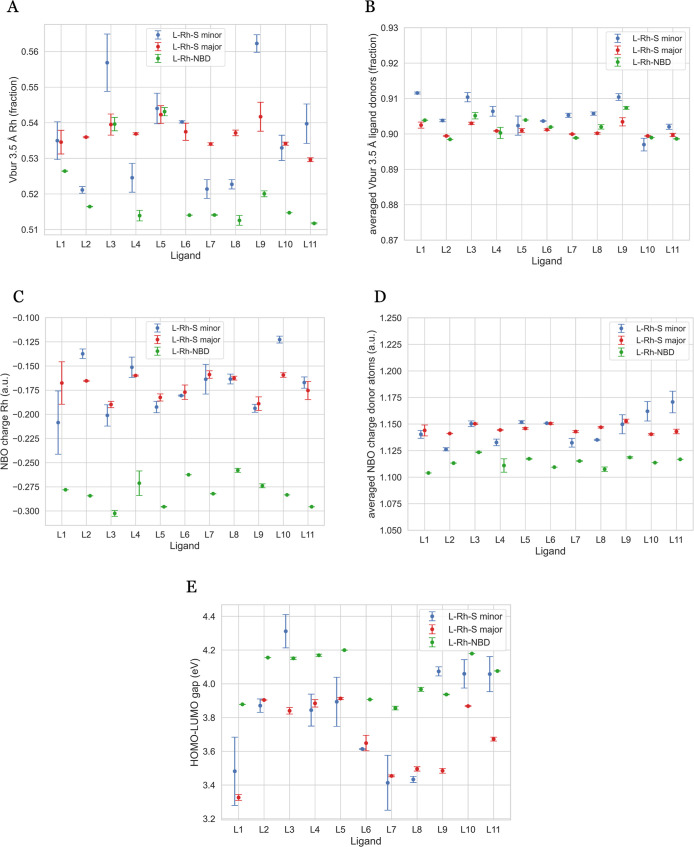
Selected descriptors Boltzmann-averaged over the conformer sets for L-Rh-S minor, L-Rh-S major, and L-Rh-NBD: the Rh NBO charge (A), donor NBO charge (B), Rh buried volume (C), donor buried volume (D), and HOMO-LUMO gap (E). Descriptors concerning the donor atoms are an average of the two P atoms.

Given the significant structural and energetic variations within the conformer ensembles, the focus now shifts to understanding their impact on the descriptors. Out of a total of 75 descriptors generated by OBeLiX,^45^ we sought to test the impact of catalyst flexibility on representative descriptors for the transition metal complexes. We have selected a subset of five descriptors for detailed analysis, including the buried volume on Rh, the buried volume on donor atoms, the NBO charge on Rh, the NBO charge on donor atoms, and the HOMO–LUMO gap. These five descriptors serve as an illustrative subset: the buried volume serves as a general steric descriptor,^58^ the NBO charge has been previously used in catalytic investigations,^26,59−61^ and the HOMO–LUMO gap represents kinetic stability.^26^Figure 7 summarizes the Boltzmann-averaged descriptor values, showing values for L-Rh-S major, L-Rh-S minor, and L-Rh-NBD, with error bars indicating standard deviations. Note that descriptor values involving donor atoms represent the average of two P atoms.

A first look at the figure reveals the varying impacts of the conformer ensembles on different descriptors. The Rh buried volume (Figure 7A), donor buried volume (Figure 7B), and charge on the donor atoms (Figure 7D) are minimally affected by the conformer ensemble, with maximum standard deviation values of 0.008, 0.003, and 0.001, respectively. Comparing the results for L-Rh-S to those for L-Rh-NBD, generally smaller standard deviations are observed with L-Rh-NBD. L4, the ligand with the highest number of conformers involving NBD, shows the largest overall variety. The plots regarding the charge on Rh (Figure 7C) and the HOMO–LUMO gap (Figure 7E) warrant independent analysis due to significant influences from (1) the conformer ensemble, (2) the initial coordination of S, and (3) the choice of the coordinating substrate.

Within a conformer set, interactions between the metal center and the substrate can significantly influence the charge on the metal center. The expectation is that the rigid substrate should manifest as a small standard deviation in the charge on Rh for the conformer ensembles of L-Rh-S. Indeed, this is seen in the maximum standard deviation of 0.03 au for the L-Rh-S minor conformer ensemble of L1. However, when different substrate coordinations are examined, significant differences emerge. With four different coordinations of S leading to four conformer sets (two L-Rh-S major and two L-Rh-S minor), a comparison of the lowest energy conformers reveals notable variations. For instance, the two lowest energy conformers of L-Rh-S major of L1 exhibit Rh NBO charge values of −0.185 and −0.180 au, while the lowest energy conformers of L-Rh-S minor of L1 exhibit values of −0.188 and −0.267 au. The difference of nearly 0.09 au between major and minor coordination conformers within the same ligand is statistically significant, given the maximum standard deviation of 0.03 au across ligands. These observations emphasize that conformer search, due to the substrate’s rigidity, has minimal impact on the metal center charge, while the initial coordination of the substrate significantly affects this descriptor value.

**Figure 8:**
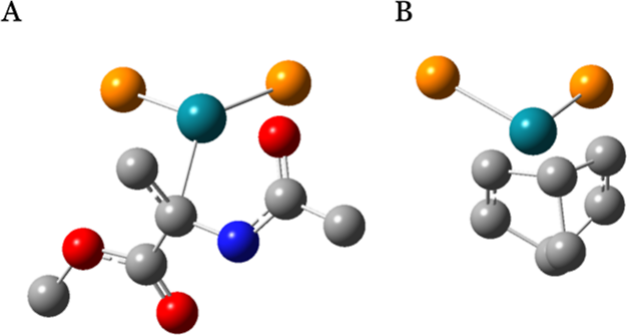
S coordination in the overall lowest energy conformer of L1-Rh-S (A) and NBD coordination in the lowest energy conformer of L1-Rh-NBD (B), with Rh NBO charge values of −0.180 and −0.278 au, respectively. For clarity, hydrogen atoms and all atoms of the bidentate ligand except P are omitted. Color coding of the atoms shows Rh (turquoise), P (orange), O (red), N (blue), and C (gray) atoms.

The metal center charge values of L-Rh-S can be compared to those of L-Rh-NBD. The highly rigid NBD molecule leads to a maximum standard deviation of only 0.01 au for ligand L4, revealing consistently lower L-Rh-NBD charge values for all ligands. The diverging descriptor values may be attributed to different substrate coordination interactions, as visualized in Figure 8. The coordination of both π-systems in the symmetric NBD structure (Figure 8B) is associated with a lower NBO charge of −0.278 au, whereas the more distorted coordination of S with the metal center (Figure 8A) leads to a higher NBO charge of −0.180 au.

**Figure 9:**
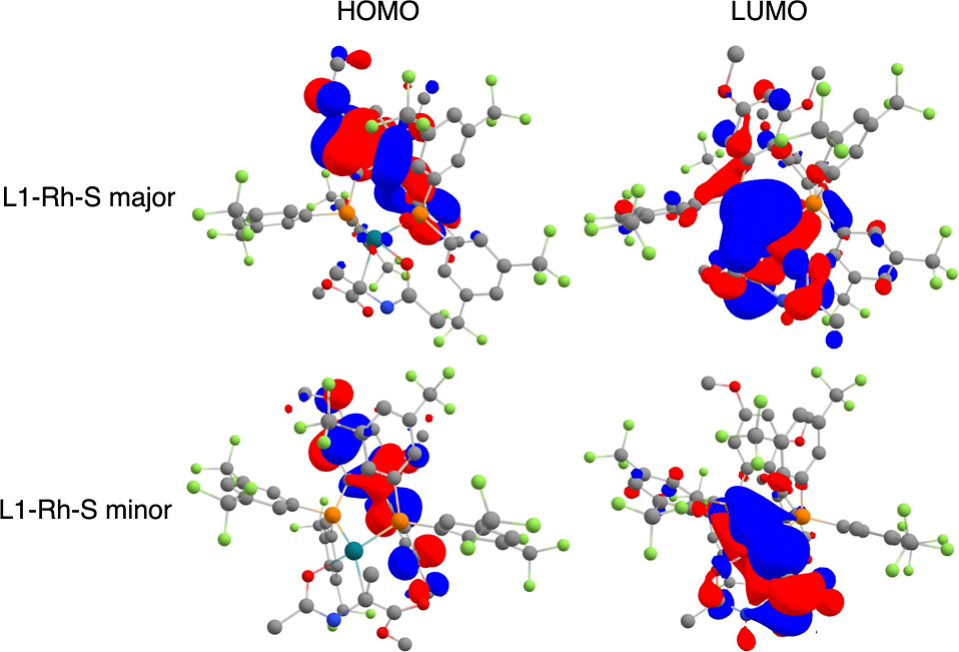
Visualization of the HOMO orbitals (left column) and LUMO orbitals (right column) for the lowest energy conformers of L1-Rh-S major (upper row) and L1-Rh-S minor (lower row). For clarity, hydrogen atoms are not shown. Color coding of the atoms shows Rh (turquoise), P (orange), O (red), N (blue), C (gray), and F (green) atoms.

Variations in the global HOMO–LUMO gap descriptor likely correlate with those of the charge on Rh. As discussed for L1, the lowest energy conformers of the four L-Rh-S coordinations show metal center charges ranging from −0.180 to −0.267 au. This variation is also reflected in diverging HOMO–LUMO gap values and visualized with the HOMO and LUMO orbitals in Figure 9. The figure illustrates different spatial distributions of HOMO orbitals (left column) and LUMO orbitals (right column) for the lowest energy conformer of L1-Rh-S major (upper row) and L1-Rh-S minor (lower row), with charges on Rh of −0.180 and −0.267 au, respectively. In L1-Rh-S major, the orbitals have more spatial overlap, reflected in a lower HOMO–LUMO gap of 3.31 eV. Conversely, L1-Rh-S minor reveals less spatial overlap and a higher HOMO–LUMO gap of 3.86 eV. With a maximum standard deviation for the HOMO–LUMO gap of 0.2 eV within a conformer ensemble, a difference exceeding 0.5 eV across different substrate coordination modes is significant, supporting previous claims that the electron distribution is sensitive to subtle conformational changes.^62^ The pronounced influence of the substrate on this global descriptor is further evidenced by the comparison of the HOMO–LUMO gaps for L-Rh-S and L-Rh-NBD (Figure 7E).

## 4 Conclusions

Herein, we present a comprehensive computational analysis of the impact of catalyst–substrate interactions on the catalyst structure and the descriptors derived from it. Focusing on a family of Rh bisphosphine catalysts (11 members), we explored a representative model catalyst system of asymmetric acetamide hydrogenation using methyl 2-acetamidoacrylate (denoted as S) as a model substrate, comparing it to the precatalyst structure with NBD. The primary objective was to study the capability of mechanistically agnostic models to capture effects that are purely substrate-specific.

A conformer search was conducted to assess the influence of substrate inclusion on the size of the generated conformer ensembles. We found that the asymmetric nature of S induces catalyst flexibility (i.e., conformational freedom within the metal–ligand system), reflected in the number of generated conformers. The maximum ensemble size involving S surpassed five times that of the maximum ensemble size involving NBD. These findings indicate that the precatalyst structure may lack critical information about the system’s flexibility, underscoring the impact of the substrate on the overall conformational landscape of the studied systems.

Delving deeper into substrate-induced catalyst flexibility, the ligand and substrate contributions were investigated separately. We unveiled that the specific substrate is rather rigid, similar to NBD. Both structures show consistently low rmsd values below or around 1 Å compared to the lowest energy conformer per conformer ensemble. The ligand, on the other hand, exhibits remarkable flexibility with rmsd values reaching up to 5 Å. This ligand flexibility challenges the traditional ‘lock-and-key’ model, supporting recent studies that highlight the importance of flexibility for achieving high selectivity and reactivity.

Finally, the influence of the found ligand flexibility was evaluated on a set of descriptors, underscoring the significance of considering the entire conformer ensemble rather than focusing solely on the most stable conformer. While structural properties showed minimal sensitivity to various conformers, electronic properties, such as the charge on Rh and the HOMO–LUMO gap, exhibited more substantial variations. More importantly, these descriptors were significantly influenced by the initially chosen substrate coordination mode. The charge on Rh can differ by almost 0.1 au depending on the substrate coordination mode, leading to a HOMO–LUMO gap difference that exceeds 0.5 eV. These discrepancies show the sensitivity of these electronic properties to the coordination environment, influenced not only by the chosen coordination but also by the specific substrate. Notably, differences in electronic properties between the substrate-specific and precatalyst structures may impact not only enantioselectivity but also conversion, suggesting that substrate inclusion may influence descriptor-based catalyst design.

The detailed analysis of substrate-specific conformer ensembles, compared with the precatalyst structure, has provided valuable insights into the catalyst–substrate interactions of a family of Rh bisphosphine catalysts. With catalyst–substrate interactions often being omitted in conventional descriptor-based catalyst design strategies, this study may offer a starting point to understand the origin of ligand flexibility and its effects on descriptors. Future investigations could build upon our findings by exploring a broader spectrum of ligands and substrates. For instance, examining another symmetric, noncyclic substrate could confirm whether substrate rigidity is an inherent property or arises from the asymmetric character of the substrate. Furthermore, integrating the substrate-specific and ensemble-averaged descriptors into data-driven catalyst design may deepen our understanding of the substrate’s significance. Such investigations may help advance the understanding of catalyst–substrate interactions in asymmetric hydrogenation, possibly contributing to more informed and effective catalyst design strategies.

## Data Availability

All inputs and outputs for DFT and CREST calculations, datasets, and code are available together with an extensive readme via 4TU.ResearchData (10.4121/ce7fb6ee-a10c-44c8-91c0-1d55d55882e3). The OBeLiX code for descriptor calculation will be available via our GitHub page (https://github.com/epics-group)

## Supporting Information Available

Selection of GFN*n*-*x*TB for conformer search; chirality and substrate coordination issues during conformer search; supporting analyses of energy vs structural differences; and origin of energy differences (PDF)
