# Supplementary material for: Impact of Model Selection and Conformational Effects on the Descriptors for In Silico Screening Campaigns: A Case Study of Rh-Catalyzed Acrylate Hydrogenation
Source: J Phys Chem C Nanomater Interfaces. 2024 May 2;128(19):7987–98. doi: 10.1021/acs.jpcc.4c01631 (PMC12025388; doi:10.1021/acs.jpcc.4c01631)
Supplement: Supplementary file 1 — jp4c01631_si_001.pdf [file jp4c01631_si_001.pdf]

Supporting Information

Impact of Model Selection and Conformational Effects on the Descriptors for In Silico Screening Campaigns: a Case Study of Rh-Catalyzed Acrylate Hydrogenation

Margareth S. Baidun ,[†,‡] Adarsh V. Kalikadien ,[†,‡] Laurent Lefort,[¶] and Evgeny A. Pidko [*,†]

†*Inorganic Systems Engineering, Department of Chemical Engineering, Faculty of Applied Sciences, Delft University of Technology, Van der Maasweg 9, 2629 HZ, Delft, The Netherlands*

‡*Contributed equally to this work*

¶*CPRD-HTE, The Janssen Pharmaceutical Companies of Johnson & Johnson, Turnhoutseweg 30, 2340 Beerse, Belgium*

E-mail: e.a.pidko@tudelft.nl

S1 Conformer search details

S1.1 Selection of GFNn-xTB for conformer search

Three GFNn-xTB methods—GFN-FF, GFN2-xTB, and the composite method GFN-FF//GFN2-xTB—were evaluated for conformer search on a representative set of ligand structures. Table S1 summarizes the results for a given input structure of L7-Rh-S. At the lower level of theory, GFN-FF generated 120 conformers within a 6 kcal/mol energy range. Due to the dense

packing of conformers, many structures will potentially converge to identical minima following DFT optimization. A notable limitation of GFN-FF was observed in the rotation of the substrate structure, leading to the generation of tetrahedral conformers from a square planar input structure (see Figure S1). Attempts to optimize tetrahedral structures with DFT resulted in convergence to a square planar form, highlighting the inaccuracy of GFN-FF for conformer search. In contrast, GFN2-xTB showed no substrate rotation issues and produced fewer conformers within the 6 kcal/mol range. However, the computational time was significantly higher, posing challenges for efficient conformer search on a larger scale.

Table S1: Summary of the conformer search results with different GFNn-xTB methods for input structure L7-Rh-S major 1. Listed are the number of generated conformers and the computational time in hours:minutes:seconds.

Method	Number of conformers	Computational time
GFN-FF	120	02:20:05
GFN2-xTB	64	24:04:41
Mix	26	02:25:10

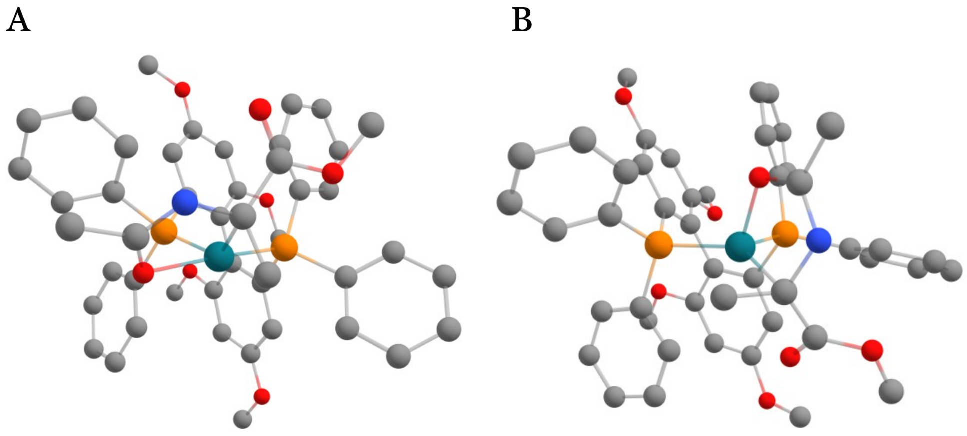

Figure S1: Comparison of the L7-Rh-S major 1 input structure (A), and the lowest energy conformer generated by CREST (B), showing the rotation of the substrate from square planar to tetrahedral coordination. For clarity, hydrogen atoms are not shown. Hydrogen atoms are omitted for clarity. Color coding of the atoms shows Rh (turquoise), P (orange), O (red), N (blue), C (grey), and F (green) atoms.

The composite GFN-FF//GFN2-xTB method significantly reduced computational time compared to GFN2-xTB, reaching a computational effort similar to that of GFN-FF. No

substrate rotation issues were observed, and fewer conformers within the specified energy range were produced. As an additional test, we applied this method to a ferrocene ligand structure. We have observed distortions in ferrocene structures when using GFN-FF. While lower energy conformers showed no distortions, some higher energy conformers exhibited slight distortions in the ferrocene structure (Figure S2). When extending the range of investigated ligands in future studies, it becomes crucial to choose a method capable of handling challenging structures like ferrocene appropriately.

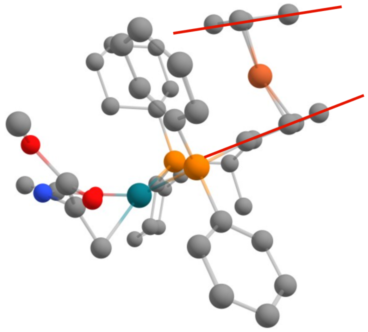

Figure S2: Distorted ferrocene structure after conformer search on an additional ferrocene ligand. The red lines highlight the distorted structure. Hydrogen atoms are omitted for clarity. Color coding of the atoms shows Rh (turquoise), P (orange), O (red), N (blue), C (grey), and F (green) atoms.

S1.2 Chirality and substrate coordination

All ligands investigated in this study exhibit axial chirality, with the R-isomer as the stereoisomer of interest. Preserving chirality throughout the conformer search process is crucial. However, using the mixed method, we observed a change in the chirality of the ligand after xtb optimization (Figure S3A and B). To address this issue, we fixed the aromatic rings on the chiral axis during both xtb optimization and conformer search, successfully yielding conformers with preserved chirality.

Another challenge arose for one ligand regarding substrate coordination, where a bond rotation caused a change in the coordination mode from minor to major (Figure S3C and D). To potentially resolve this issue, we contemplated constraining certain (dihedral) angles

between the substrate, metal center, and donor P atoms. However, recognizing that applying these constraints uniformly across all ligands would constrain the conformer search for each ligand, we decided to exclude this particular ligand from the study entirely. Consequently, the contemplated fixation of angles was not implemented on the ligands in this work.

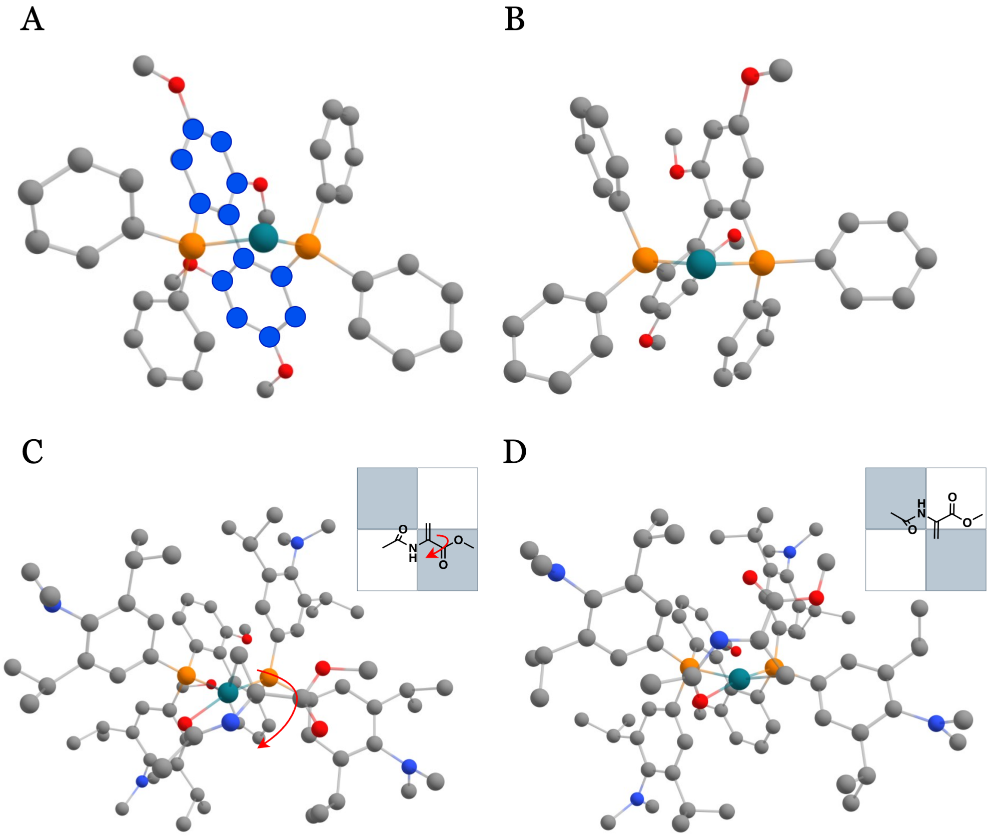

Figure S3: Distorted chirality and substrate coordination following conformer search. To maintain the desired (R)-chirality, the atoms indicated in blue must be fixed in space (A). When no atoms are fixed, CREST occasionally generates the (S)-isomer (B). One ligand was discarded from the study, due to change of substrate coordination from L-Rh-S minor in the CREST input (C) to L-Rh-S major (D). The red arrow in the input structure indicates the substrate bond rotation leading to coordination change. Hydrogen atoms are omitted for clarity. Color coding of the atoms shows Rh (turquoise), P (orange), O (red), N (blue), C (grey), and F (green) atoms.

S2 Energy vs structural differences

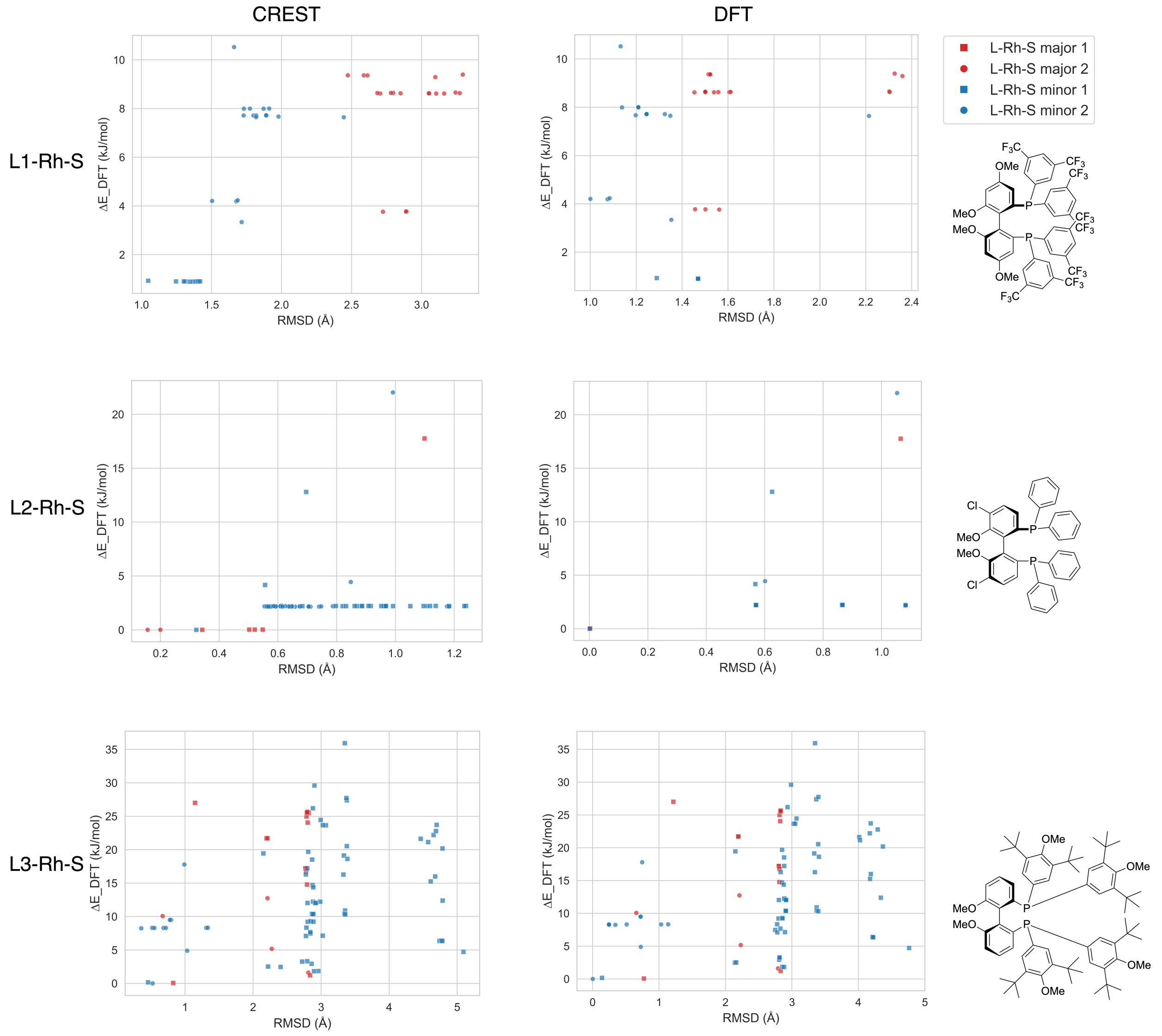

Figure S4: DFT-based energies plotted against structural variation for the conformer ensembles of ligands L1-Rh-S (upper row), L2-Rh-S (middle row), and L3-Rh-S (lower row). The RMSD values are calculated on structures obtained from CREST (left column) or DFT (right column). Within each conformer set, the conformer with the lowest DFT-based energy is taken as reference point for the ΔE and RMSD values.

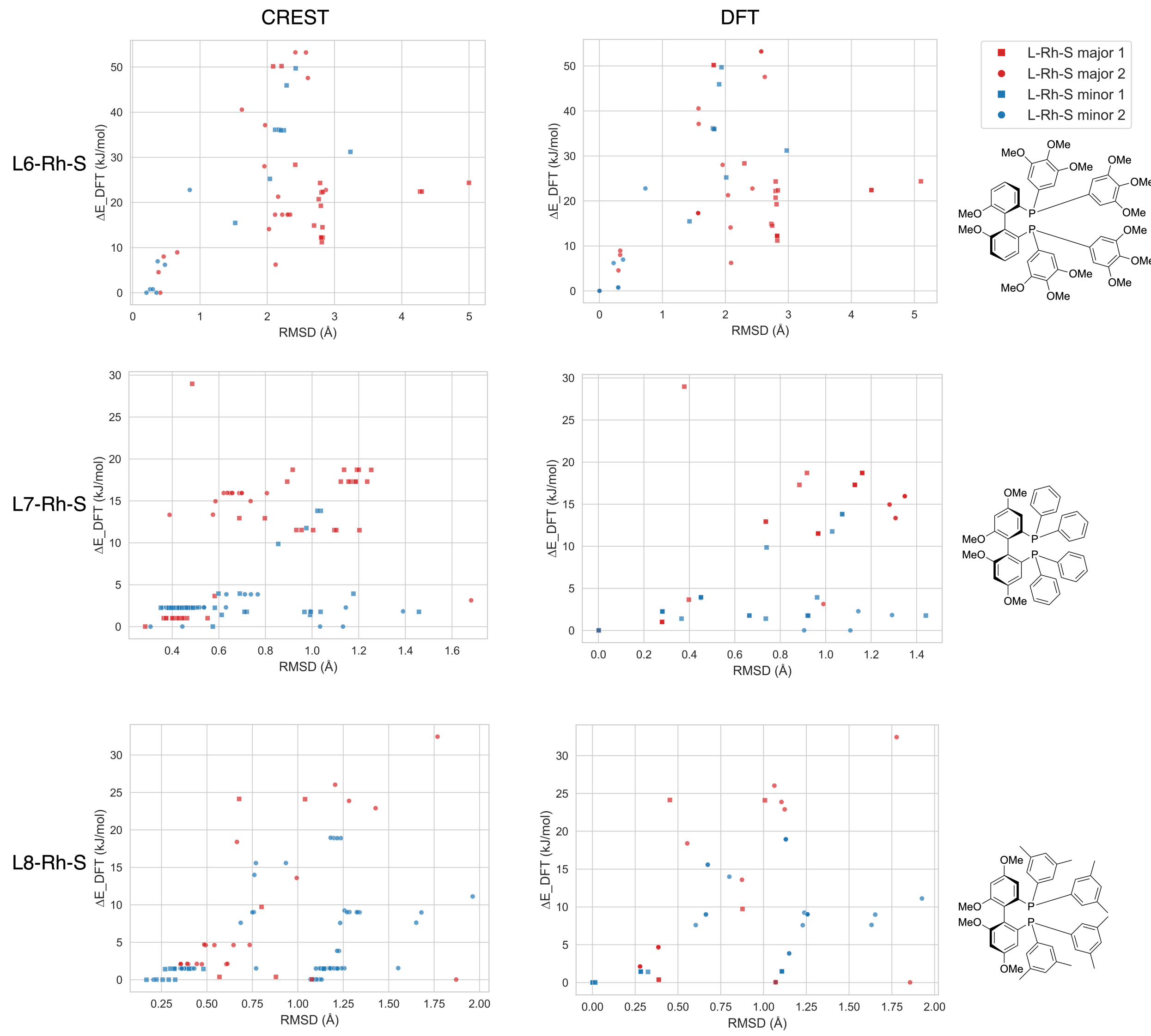

Figure S5: DFT-based energies plotted against structural variation for the conformer ensembles of ligands L6-Rh-S (upper row), L7-Rh-S (middle row), and L8-Rh-S (lower row). The RMSD values are calculated on structures obtained from CREST (left column) or DFT (right column). Within each conformer set, the conformer with the lowest DFT-based energy is taken as reference point for the ΔE and RMSD values.

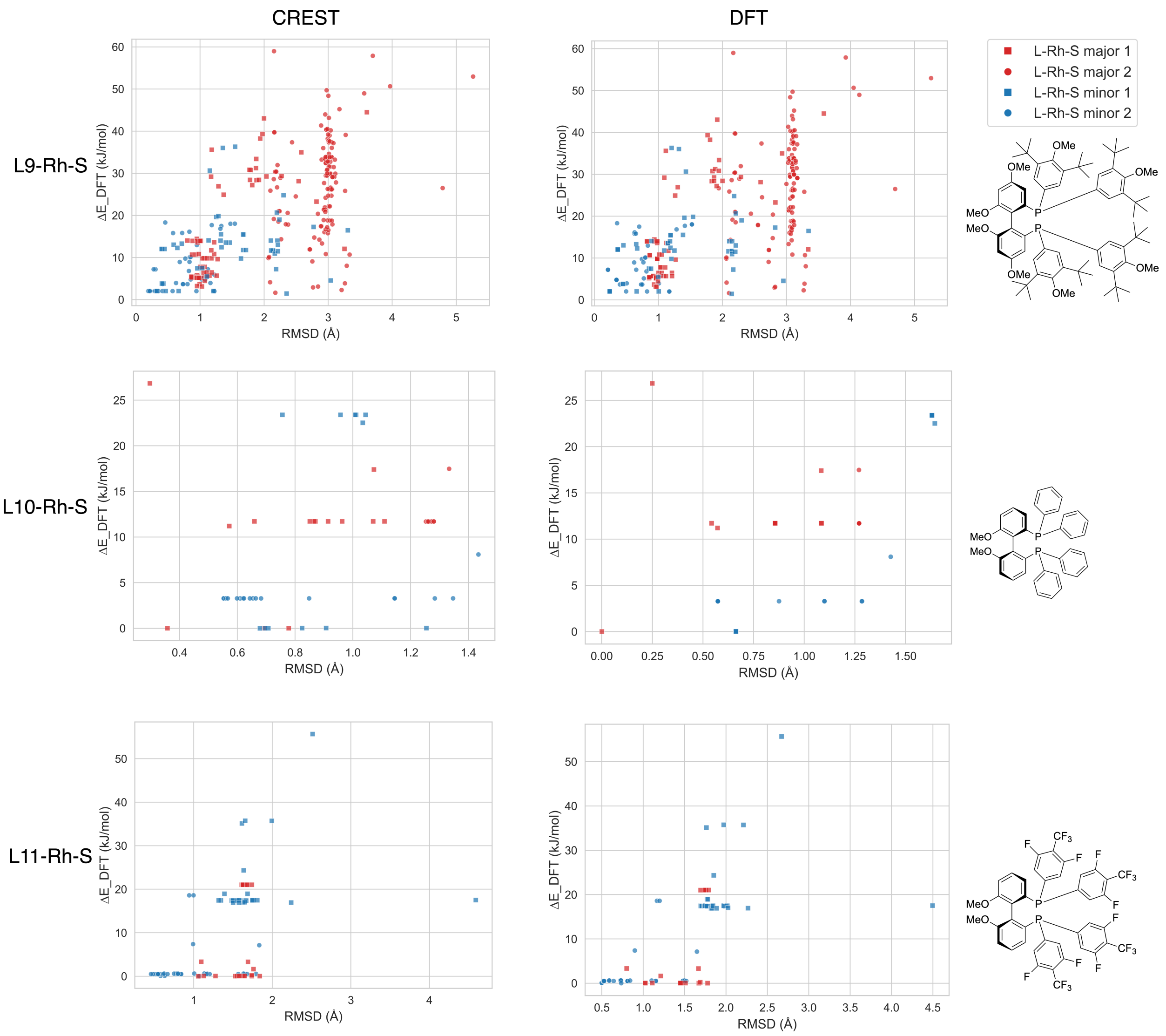

Figure S6: DFT-based energies plotted against structural variation for the conformer ensembles of ligands L9-Rh-S (upper row), L10-Rh-S (middle row), and L11-Rh-S (lower row). The RMSD values are calculated on structures obtained from CREST (left column) or DFT (right column). Within each conformer set, the conformer with the lowest DFT-based energy is taken as reference point for the ΔE and RMSD values.

Figures S4, S5, and S6 depict the structural differences of the generated L-Rh-S conformers for L1-L3, L6-L8, and L9-L11, respectively. In each subplot, the y-axis contains the DFT-based energy of the conformers relative to the lowest energy conformer within each conformer set. The x-axis shows the structural differences, represented by the RMSD value, compared to the lowest energy conformer within each conformer set. The RMSD values are based either

on CREST conformer structures (left column) or DFT-optimized conformer structures (right column).

For L1, L2, L7, L8, and L10, numerous CREST conformers converge to identical minima following DFT optimization. The most significant difference between conformers identified by CREST and DFT is observed in L2, where 73 conformers identified by CREST converge to only 12 distinct minima afer DFT optimization. In contrast, L3, L6, L9, and L11 show structural differences identified by CREST that are preserved after DFT optimization. L9 stands out with the largest number of conformers, with the 299 identified conformers converging into well over 200 minima after DFT optimization. Of the 11 investigated ligands, 5 exhibit conformer ensembles preserved after DFT optimization, while 6 show ensembles converging into fewer distinct structures. These findings emphasize the importance of DFT optimization after conformer search, even though CREST-generated conformer ensembles provide some insights into the system's flexibility.

Similar to the results discussed in the paper, Figures S4, S5 and S6 reveal instances of DFT-optimized structures (right column in the figures) with varying RMSD values but minimal energetic differences. For exampel, the L1-Rh-S major 2 ensemble shows a group of structures in a narrow ΔE range around 8.5 kJ/mol and RMSD values ranging from 1.45 to 2.30 Å. Conversely, the conformer ensemble of L9-Rh-S major 2 exhibits a narrow vertical strip of conformers around the RMSD value of 3 Å, with energies ranging from 13 to 48 kJ/mol. Both instances, featuring conformer groups similar in either ΔE or RMSD value, may yield diverging descriptor values, significantly affect descriptor-based predictions, thus emphasizing the importance of careful conformer consideration.

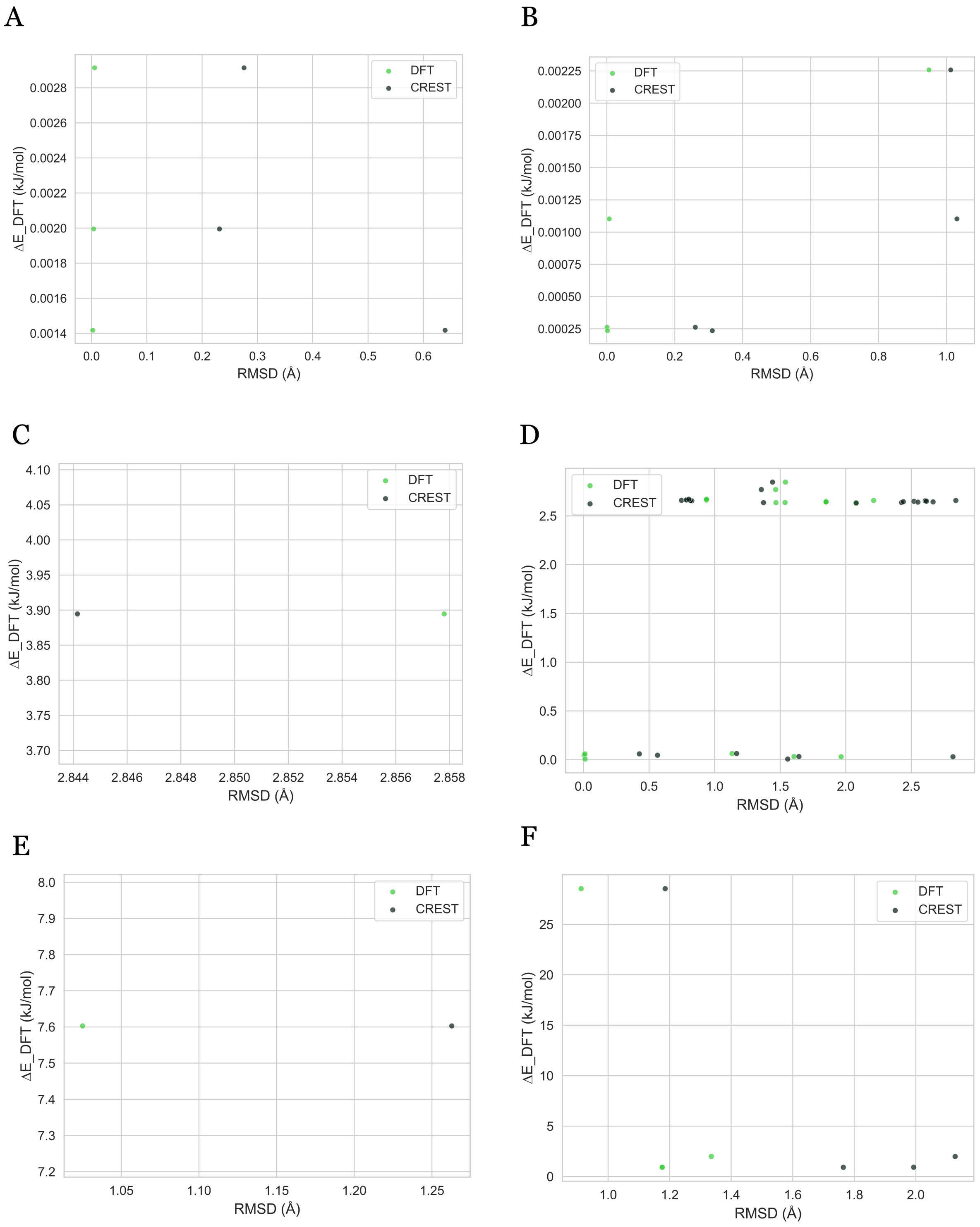

Figure S7: DFT-based energies plotted against structural variation for the conformer ensembles of L1-Rh-NBD (A), L2-Rh-NBD (B), L3-Rh-NBD (C), L4-Rh-NBD (D), L5-Rh-NBD (E), and L7-Rh-NBD (F). The RMSD values are calculated on structures obtained from CREST or DFT, indicated by different colors. The conformer with the lowest DFT-based energy is taken as a reference point for the ΔE and RMSD values.

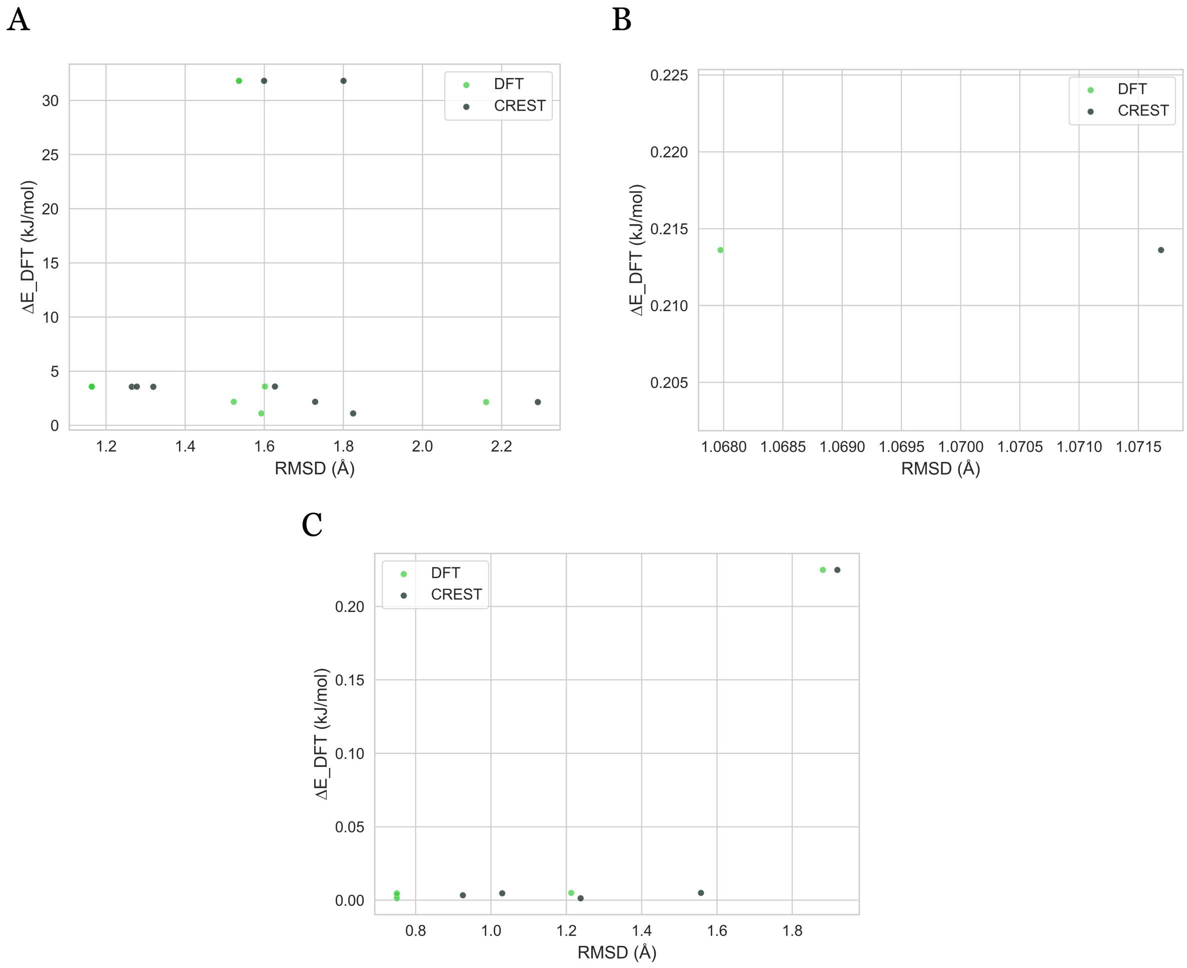

Figure S8: DFT-based energies plotted against structural variation for the conformer ensembles of ligands L8-Rh-NBD (A), L9-Rh-NBD (B), and L11-Rh-NBD (C). The RMSD values are calculated on structures obtained from CREST or DFT, indicated by different colors. The conformer with the lowest DFT-based energy is taken as a reference point for the ΔE and RMSD values.

Figures S7 and S8 illustrate the structural differences in the generated L-Rh-NBD conformers for L1-L5, L7, and L8, L9, L11, respectively. Note that L6 and L10 are not included due to the generation of only one conformer. The y-axis displays the DFT-based energy of the conformers compared to the lowest energy conformer for each ligand. The x-axis shows the RMSD values compared to the lowest energy conformer. The RMSD calculation is performed either on structures obtained from CREST, or on DFT-optimized structures, indicated by the different colors.

Based on the work of Krieger and Pidko, ΔE of 5 kJ/mol is considered as a threshold value above which conformers are considered significantly different in energy.[1] Despite structural differences being present in the conformer ensembles, the majority of L-Rh-NBD conformers show DFT-optimized structures with ΔE values below this threshold value. Only ligands L5, L7, and L8 show outlier structures, with the maximum ΔE value for L8 exceeding 30 kJ/mol. These findings highlight the difference in conformer behavior of L-Rh-S compared to L-Rh-NBD.

S3 Energetic differences

Given the discussions suggesting that the ligand causes a broad RMSD range, we attempted to investigate the origin of energetic differences within the conformer ensembles as well. This was done by analyzing structures with similar energetic values but different RMSD values (the "horizontal" comparison) and structures with similar RMSD values but different energetic values (the "vertical" comparison). Note that in the vertical comparison, similar RMSD values indicate that the structures are "similarly different" from the lowest energy conformer, not necessarily similar to each other. These horizontal and vertical analyses aim to clarify the nature of structural changes that lead to significant discrepancies in either RMSD or energy values.

The vertical and horizontal comparison can be visualized by a structural overlay. The horizontal overlay shown in Figure S9 reveals ligand variations, particularly in the rotation of the tBu groups, while the substrate remains unchanged. For these two structures, the difference in RMSD and ΔE values is 2.30 Å and 0 kJ/mol. The vertical overlay reveals more substantial structural differences, with a crucial distinction being the rotation of the COOMe group in the substrate. For the illustrated conformers, the difference in RMSD and ΔE values is 0.03 Å and 22 kJ/mol.

To assess the contribution of each part to the structural and energetic differences, we

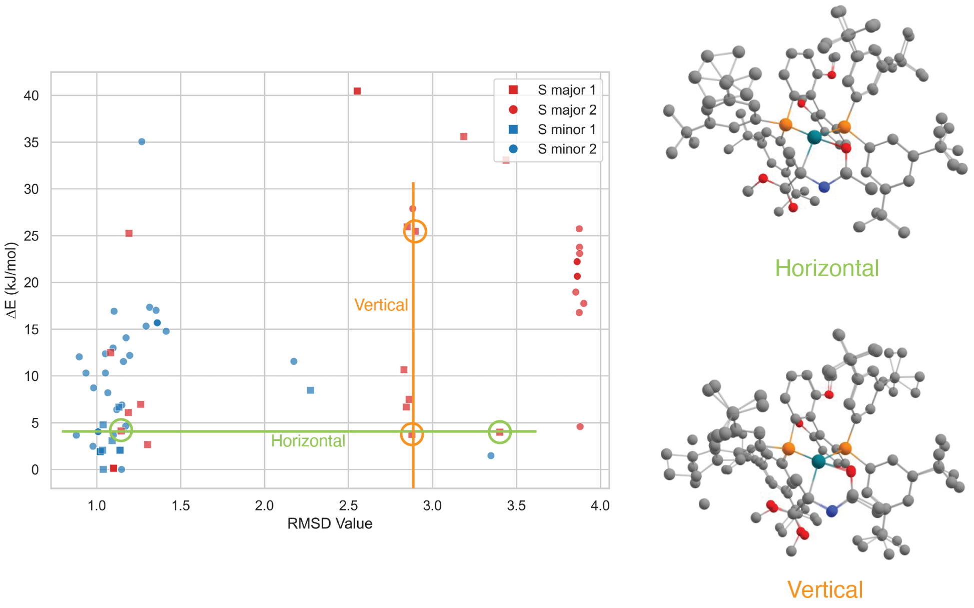

Figure S9: Structural overlay of two conformers with diverging RMSD values and constant ΔE value (horizontal), and two conformers with similar diverging ΔE values and constant RMSD value (vertical). Structures are taken as en example from the data of L5-Rh-S.

examined separate ligand and substrate RMSD differences. It is important to note that the range of substrate RMSD values is often significantly smaller than in the ligand case. Therefore, differences are expressed as percentages relative to the largest ligand/substrate RMSD value in the conformer set. In the horizontal comparison of Figure S9, the change in ligand RMSD is 66.2% of the largest ligand RMSD value in the conformer set, while the substrate RMSD difference is only 0.005% of the largest substrate RMSD value. The vertical comparison reveals a contrasting trend, with a ligand difference of 1.9% and a substrate difference of 98.9%. Similar analyses for various conformers with extreme RMSD and ΔE values are summarized in Figure S10. In general, the ligand contribution is dominant in the horizontal comparisons, while the substrate contribution is dominant in the vertical comparisons. These results underscore that large structural changes caused by the ligand do not necessarily translate to significant energetic variations, while small changes in the substrate can lead to substantial energetic differences.

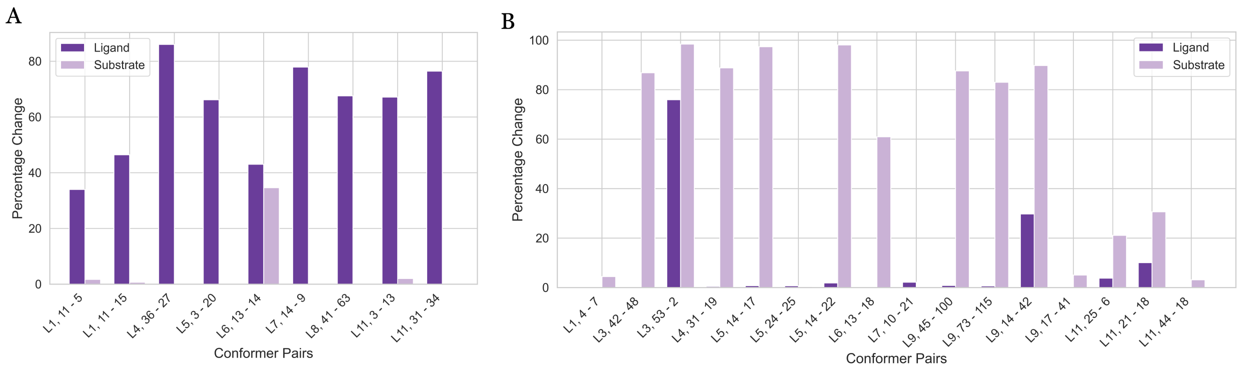

Figure S10: Structural comparisons of diverging RMSD with constant ΔE (A), and diverging ΔE with constant RMSD (B). For each structural comparison, separate ligand RMSD and substrate RMSD values are compared. These are indicated as percentages relative to the maximum ligand/substrate RMSD value within the conformer set.

References

(1) Krieger, A. M.; Pidko, E. A. The Impact of Computational Uncertainties on the Enantioselectivity Predictions: A Microkinetic Modeling of Ketone Transfer Hydrogenation with a Noyori-type Mn-diamine Catalyst. *ChemCatChem* **2021**, *13*, 3517–3524.
